# Sequencing and analysis of the complete mitochondrial genome of *Crocidura tanakae* from China and its phylogenetic analysis

**DOI:** 10.1080/23802359.2019.1659118

**Published:** 2019-08-29

**Authors:** Jin Zhi-Min, Qin Ke-Song, Li Bo-Qi, Zhang Jun-Sheng, Cai He, Li Jin-Xun, Wang Zhu, Liu Zhu

**Affiliations:** College of Life Science and Technology, Mudanjiang Normal University, Mudanjiang, People’s Republic of China

**Keywords:** Control region, mitogenome, phylogenetic trees, *Crocidura tanakae*

## Abstract

The complete mitogenome sequence of *Crocidura tanakae* was determined using long PCR. The genome was 16,969 bp in length and contained 13 protein-coding genes, 2 ribosomal RNA genes, 22 transfer RNA genes, 1 origin of L strand replication and 1 control region. The overall base composition of the heavy strand is A (32.5%), C (22.3%), T (31.9%), and G (13.3%). The base compositions present clearly the A–T skew, which is most obviously in the control region and protein-coding genes. Mitochondrial genome analyses based on MP, ML, NJ, and Bayesian analyses yielded identical phylogenetic trees. The five *Crocidura* species formed a monophyletic group with the high bootstrap value (100%) in all examinations. This study verifies the evolutionary status of *C. tanakae* in Soricidae at the molecular level. The mitochondrial genome would be a significant supplement for the *C. tanakae* genetic background.

In this article, the complete mitochondrial genome of *Crocidura tanakae* was sequenced for the first time on ABI 3730XL using a primer walking strategy and the long and accurate PCR, with five pairs of long PCR primers and with 14 pairs of sub-PCR primers. A muscle sample was obtained from a female *C. tanakae* captured from Bijie regions of Wumeng Mountains in Guizhou Province, China (26°24′22″ N, 105°44′04″ E). The muscle tissue was preserved in 95% ethanol and stored at −75 °C before use. The specimen and its DNA are stored in Animal and Plant Herbarium of Mudanjiang Normal University. The voucher number is GZ2019004.

The mitochondrial genome is a circular double-stranded DNA sequence that is 16,969 bp long including 13 protein-coding genes, 2 rRNA genes, 22 tRNA genes, one origin of L strand replication, and one control region. The accurate annotated mitochondrial genome sequence was submitted to GenBank with accession number MN128390. The arrangement of the multiple genes is in line with other Talpidae species (Mouchaty et al. 2000; Nikaido et al. 2003; Cabria et al. 2006; Hou et al. 2016; Xu et al. 2016; Gutiérrez et al. 2018; Jia et al. 2018) and most mammals (Nikaido et al. 2001; Fontanillas et al. 2005; Meganathan et al. 2012; Yoon et al. 2013; Xu et al. 2012, 2013; Kim et al. 2013, 2017; Huang et al. 2014, 2016; Xu et al. 2016; Liu et al. 2016; Liu, Tian, Jin, Jin, et al. 2017; Liu, Tian, Jin, Dong, et al. 2017; Liu, Wang, et al. 2017; Liu et al. 2018; Liu, Dang, et al. 2019; Liu, Qin, et al. 2019; Jin et al. 2017).

The control region of *C. tanakae* mitochondrial genome was located between the tRNA-Pro and tRNA-Phe genes, and contains only promoters and regulatory sequences for replication and transcription, but no structural genes. Three domains were defined in the large mole mitochondrial genome control region (Zhang et al. 2009): the extended termination-associated sequence (ETAS) domain, the central conserved domain (CD), and the conserved sequence block (CSB) domain.

The total length of the protein-coding gene sequences was 11,415 bp. Most protein-coding genes initiate with ATG except for ND2, ND3, and ND5, which began with ATC or ATT. Six protein-coding genes terminated with TAA whereas the *Cyt b* gene terminated with AGA. The incomplete stop codons (T–– or TA–) were used in ND1, ND3, COX3, ATP6, and ND4. A strong bias against A at the third codon position was observed in the protein-coding genes. The frequencies of CTA (Leu), ATT (Ile), TTA (Leu), and ATA (Met) were higher than those of other codons. The length of *tRNA* genes varied from 57 to 75 bp.

Most *C. tanakae* mitochondrial genes were encoded on the H strand, except for the *ND6* gene and eight *tRNA* genes, which were encoded on the L strand. Some reading frame intervals and overlaps were found. One of the most typical was between ATP8 and ATP6. The L-strand replication origin (OL) was located within the WANCY region containing five *tRNA* genes (*tRNATrp*, *tRNA-Ala*, *tRNA-Asn*, *tRNA-Cys*, *tRNA-Tyr*). This region was 37-bp long and had the potential to fold into a stable stem-loop secondary structure. The total base composition of *C. tanakae* mitochondrial genome was A (32.5%), C (22.3%), T (31.9%), and G (13.3%). The base compositions clearly present the A-T skew, which was most obviously in the control region and protein coding genes.

To explore the evolution of Insectivora shrews which include Soricidae and Talpidae, especially the evolution of genus *Crocidura* from China, here, we investigate the molecular phylogenetics of Chinese *C. tanakae* using complete mitochondrial genome sequence of 36 species. All sequences generated in this study have been deposited in the GenBank (Figure 1).

**Figure 1.**
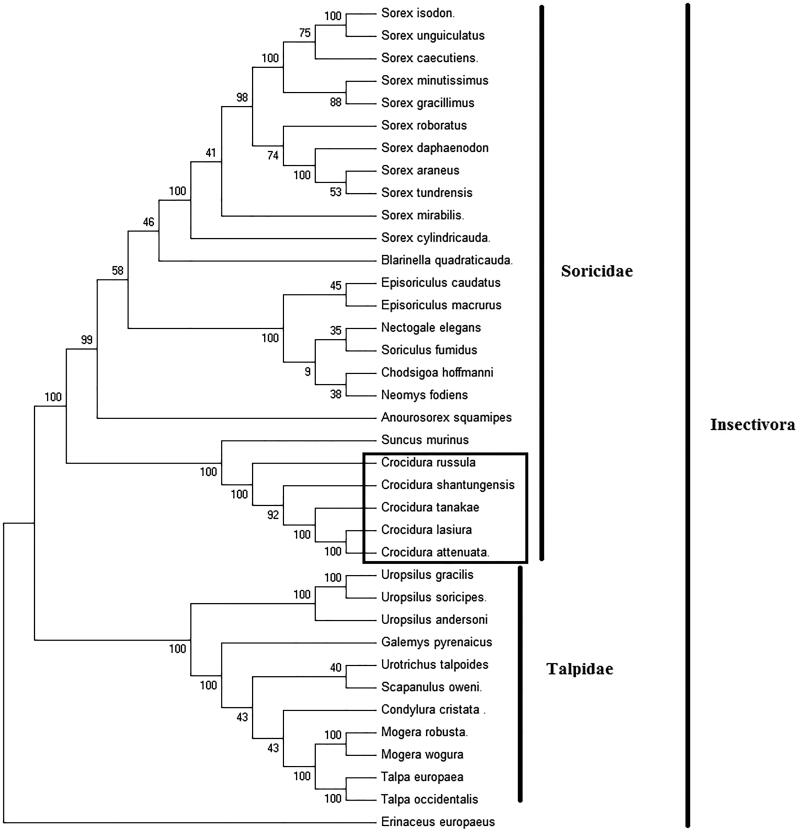
Phylogenetic tree generated using the Maximum Parsimony method based on complete mitochondrial genomes. *Chodsigoa hoffmanni* (MK940327), *Crocidura tanakae* (MN128390), *Crocidura lasiura* (KR007669), *Crocidura shantungensis* (JX968507), *Crocidura attenuata* (KP120863), *Crocidura russula* (AY769264), *Episoriculus macrurus* (KU246040), *Episoriculus caudatus* (KM503097), *Neomys fodiens* (KM092492), *Nectogale elegans* (KC503902), *Anourosorex squamipes* (KJ545899), *Blarinella quadraticauda* (KJ131179), *Suncus murinus* (KJ920198), *Soriculus fumidus* (AF348081), *Sorex araneus* (KT210896), *Sorex cylindricauda* (KF696672), *Sorex unguiculatus* (AB061527), *Sorex tundrensis* (KM067275), *Sorex caecutiens* (MF374796), *Sorex roboratus* (KY930906), *Sorex isodon* (MG983792), *Sorex gracillimus* (MF426913), *Sorex mirabilis* (MF438265), *Sorex daphaenodon* (MK110676), *Sorex minutissimus* (MH823669), *Talpa europaea* (Y19192), *Urotrichus talpoides* (AB099483), *Uropsilus soricipes* (JQ658979), *Uropsilus gracilis* (KM379136), *Mogera wogura* (AB099482), *Mogera robusta* (MK431828), *Condylura cristata* (KU144678), *Galemys pyrenaicus* (AY833419), *Scapanulus oweni* (KM506754), *Talpa occidentalis* (MF958963), *Uropsilus andersoni* (MF280389), and *Erinaceus europaeus* (NC002080).

Mitochondrial genome analyses based on MP, ML, NJ, and Bayesian analyses yielded identical phylogenetic trees, indicating a close phylogenetic affinity of shrews. The phylogram obtained from Maximum Parsimony method is shown in Figure 1. It shows that two major phyletic lineages were present in Insectivora: Soricidae and Talpidae. Soricidae comprised *Chodsigoa hoffmanni, C. tanakae, Crocidura lasiura*, *Crocidura shantungensis*, *Crocidura attenuata*, *Crocidura russula*, *Episoriculus macrurus*, *Episoriculus caudatus*, *Neomys fodiens*, *Nectogale elegans*, *Anourosorex squamipes*, *Blarinella quadraticauda*, *Soriculus fumidus*, *Suncus murinus*, *Sorex araneus*, *Sorex tundrensis*, *Sorex caecutiens*, *Sorex roboratus*, *Sorex isodon*, *Sorex gracillimus*, *Sorex mirabilis*, *Sorex cylindricauda, Sorex unguiculatus, Sorex daphaenodon*, and *Sorex minutissimus* was supported by bootstrap values of 100%. Talpidae comprised *Talpa europaea*, *Urotrichus talpoides*, *Mogera wogura*, *Condylura cristata*, *Uropsilus soricipes*, *Mogera robusta*, *Galemys pyrenaicus*, *Uropsilus gracilis, Talpa occidentalis, Uropsilus andersoni,* and *Scapanulus oweni* was supported by bootstrap values of 100%. The five *Crocidura* species formed a monophyletic group with the high bootstrap value (100%) in all examinations. This study verifies the evolutionary status of *C. tanakae* in Soricidae at the molecular level. The mitochondrial genome would be a significant supplement for the *C. tanakae* genetic background.
